# In‐Gel Direct Laser Writing for 3D‐Designed Hydrogel Composites That Undergo Complex Self‐Shaping

**DOI:** 10.1002/advs.201700038

**Published:** 2017-07-25

**Authors:** Akihiro Nishiguchi, Ahmed Mourran, Hang Zhang, Martin Möller

**Affiliations:** ^1^ DWI Leibniz‐Institute for Interactive Materials RWTH Aachen University Forckenbeck str. 50 D‐52056 Aachen Germany

**Keywords:** actuators, biomimetics, hydrogels, multiphoton lithography, stimuli‐responsive materials

## Abstract

Self‐shaping and actuating materials inspired by biological system have enormous potential for biosensor, microrobotics, and optics. However, the control of 3D‐complex microactuation is still challenging due to the difficulty in design of nonuniform internal stress of micro/nanostructures. Here, we develop in‐gel direct laser writing (in‐gel DLW) procedure offering a high resolution inscription whereby the two materials, resin and hydrogel, are interpenetrated on a scale smaller than the wavelength of the light. The 3D position and mechanical properties of the inscribed structures could be tailored to a resolution better than 100 nm over a wide density range. These provide an unparalleled means of inscribing a freely suspended microstructures of a second material like a skeleton into the hydrogel body and also to direct isotropic volume changes to bending and distortion motions. In the combination with a thermosensitive hydrogel rather small temperature variations could actuate large amplitude motions. This generates complex modes of motion through the rational engineering of the stresses present in the multicomponent material. More sophisticated folding design would realize a multiple, programmable actuation of soft materials. This method inspired by biological system may offer the possibility for functional soft materials capable of biomimetic actuation and photonic crystal application.

Active movement in biological systems such as bending of plants, snapping of prestressed structures like in the Venus flytrap, and the ciliary motion in microorganisms, is regulated by spatially ordered micro‐ and nanostructures.^1^ For example, pine cones open and close by means of anisotropic multilayer structures in response to humidity, where local stress is generated by constrained volume changes in response to humidity.^2^ These and other examples have inspired the development of mechanically active, stimuli‐responsive materials.^3^ Particularly prominent are hydrogel materials that can be stimulated to undergo a volume phase transition.^4^ This shape changing capability has been employed for soft actuators and artificial muscles.^5^, ^6^, ^7^, ^8^, ^9^ The volume change can be transformed into bending and distortion modes either in bi‐ or multilayer constructions^5^ or as we have shown recently by nonequilibrium actuation.^6^ A bilayer structure presents the most simple case of an anisotropic design, where the volume change of the constituent components causes a stress that can result in large amplitude bending, folding, and buckling and where the motion can become very fast.^6^, ^10^ Rapid nastic motion in plants, for example, as observed for mimosa, the Venus fly trap, or seed and pollen dispersal is typically based on much more complex structures.^1^, ^2^, ^10^ Although 2D soft lithography, printing, and self‐assembly techniques can be used to fabricate bilayers, 2D patterns, and even gradients in thickness and crosslinking,^11^, ^12^, ^13^, ^14^, ^15^, ^16^, ^17^, ^18^ it is unquestionable that the actuation and control of complex motion as it is desired for soft matter microrobots as well as for devices that mix, sort, and circulate fluids will require even further structural control in all three dimensions.^1^ Already the relatively slow and basic opening and closing motion of a pine cone relies on a structural architecture that can be barely prepared by self‐assembly or 2D lithography.^2^

Although it remains a challenge to combine materials that are significantly distinguished in their mechanical properties, 3D printing and 3D lithography techniques provide new means to assemble heterogeneous compositions to a complex 3D structure, that fulfill the requirements for actuation of controlled motion. Fast hydraulic actuation requires small, micrometer‐scale heterostructures that are sufficiently open in order to allow the uptake and release of water in short time. Structures of this size range can be crafted by multiphoton lithography (MPL).^19^, ^20^, ^21^, ^22^ Due to the optical nonlinearity of multiphoton absorption and the intensity threshold for photopolymerization, curing of a photoresist can be confined within the focal volume of a laser beam (ellipsoidal voxels). This maskless and free‐form lithography, also known as direct laser writing (DLW), extends the microfabrication to spatial resolutions smaller than 200 nm. Emerging applications are preparation of photonic crystals,^23^ metamaterials,^24^ and ceramic nanostructures.^25^ For soft hydrogel structures, DLW has been reported to deposit protein microstructures within hyaluronic acid gels to direct nerve growth;^26^ to crosslink bovine serum albumin (BSA) in layers with varying stiffness in order to enable pH‐sensitive bending,^27^ and to prepare hydrogel patches on a field of microscopic epoxy pillars.^28^ 3D‐printing system also allows for the fabrication of soft actuators with controlled motions.^29^, ^30^ In the latter case, contraction of the hydrogels caused concentric bending of the pillars. Here we report on the preparation of hydrogels that contain freely suspended solid microstructures of a second stiff material like a skeleton that can direct the swelling/deswelling to a controlled bending and distortion. We will address the problem that DLW of soft structures such as hydrogels suffers from the fact that the contours become blurred at the expense of spatial resolution,^25^, ^26^, ^28^ and how a second material component can be introduced by simple means on the micrometer scale. We will demonstrate the principally huge variability in the structural design and control of the local composition.

The key aspect of our approach is, what we call in‐gel direct laser writing (in‐gel DLW), a technique depicted in **Figure** **1**. A hydrogel is prepared by means of a poly(perfluorether) mold with an appropriate shape and crosslinking density (see also Figure S4a, Supporting Information).^31^ This network is swelled with monomeric photoresist, and a freely designed structure is directly written into the gel structure. If the photoresist monomer is selected to yield a more rigid polymer, that phase separates from the hydrogel, stiff structural units can be prepared inside the hydrogel, that can direct volume changes to become anisotropic (Figure 1a). Compared to the inverse procedure where the rigid structure is generated first and the hydrogel is filled in by impregnation with monomer and subsequent polymerization, in‐gel DLW, promises distinct advantages: (i) The gel may suppress convection, and the spatial resolution may be improved, (ii) in situ formation of the rigid skeleton circumvents problems with the collapse of the structures that may occur during drying due to the shrinkage of materials and surface tension,^32^, ^33^ and (iii) the skeleton structures can be freely suspended within the hydrogel and are not necessarily linked to a substrate. In‐gel DLW of free suspended elements will also allow preparing different structures that are not connected to each other, so that the general compliance of the hydrogel matrix is directed but not intrinsically reduced. In principle but beyond the scope of this paper, the swelling technique will also allow inscription of different materials, respectively, elasticity into the hydrogel body.

**Figure 1:**
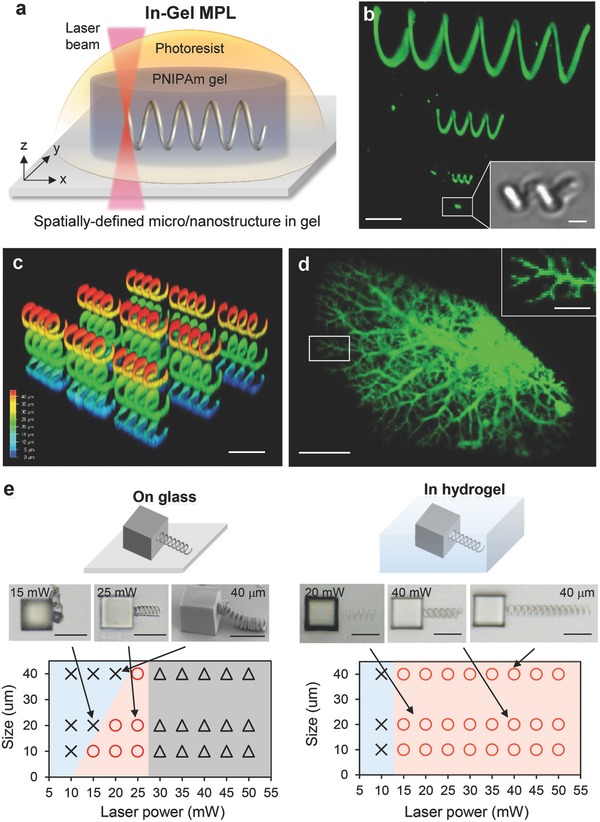
a) Schematic illustration of in‐gel DLW process. DLW based on two‐photon polymerization was performed in PNIPAm hydrogel preswelled with an acrylic photoresist. The feature size of 3D‐micro/nanostructures with controlled shape, 3D position, and stiffness can be fabricated in hydrogels. b) 3D‐reconstructed CLSM image of helical structures with different diameter and length (φ–*l*: 30–120, 10–40, 5–10, 1–2 µm) in hydrogel. The 30 mW of laser power and 5000 µm s^−1^ of scan speed was used for writing, and the slicing/hatching distance was fixed at 200 nm. Inset: phase contrast image. c) 3D‐reconstructed CLSM image of helical structures with 10 µm spacing in PNIPAm hydrogel. The intensity map denotes the height of the structures from the bottom. d) 3D‐reconstructed CLSM image of blood vessel model structure. e) Shape diagram of helices written on a glass and in hydrogel. The laser power and helix size were varied from 10 to 50 mW and from 10 to 40 µm, respectively. Scan speed was fixed at 5000 µm s^−1^. The slicing/hatching distance was fixed at 200 nm. Circle, cross, and triangle marks denote the formation of structure, no structure was formed, and polymerization proceeded but the structure was collapsed by bubble formation, respectively. Insets: phase contrast images. Scale bars, 20 µm for (b)–(e), 500 nm for insets of (b) and (d).

So far we realized this concept with thermoresponsive poly(*N*‐isopropylacrylamide) (PNIPAm) gels that were swelled by acrylate monomers as the photoresist (IP‐L 780, refractive index of photoresist, *n*: 1.48, Nanoscribe GmbH, Germany^34^) containing an α‐aminoketone as multiphoton initiator. The uptake of IP‐L yielded an equilibrium degree of swelling by a factor of 1.2 in area, corresponding to a volume increase by 30%. For comparison, the swelling in water resulted in a volume expansion by a factor >8. The IPL‐780 swelled PNIPAm film was placed on a Nanoscribe MPL device with femtosecond laser (emission wavelength: 780 nm), 3D‐piezo scanning stage, and oil‐immersion 63× objective lens (NA: 1.4) and the helix structure was inscribed by localized two‐photon initiation of the polymerization of IP‐L 780. After inscribing the helix structure, the remaining photoresist was extracted by acetone, and the gel was swelled in ultrapure water, resulting in further volume expansion. Figure 1b,c depicts the confocal laser scanning microscopy (CLSM) image of an acrylate helix that has been inscribed into a PNIPAm gel film with 50 µm of thickness. This way IP‐L 780 resin helices of different diameter and length (φ–*l*: 30–120, 10–40, 5–10, 1–2 µm) were fabricated as freely suspended objects within the PNIPAm gel (Figure 1b). Figure 1c demonstrates the formation of a 3D array of disconnected helices. In gel DLW also allowed the fabrication of very fragile and nonperiodic structures without any collapse and support by solid substrate. This is demonstrated by the tree‐like branched structure in Figure 1d, which is a spatial reconstruction of the capillary branching of blood vessels. Remarkably, the smallest feature sizes, which we could achieve, were in the range of tens of nanometers (Figure 1d). In principle, the spatial resolution of MPL is determined by the focal volume of the laser beam, that is, the lateral and axial radii of 150 and 300 nm, respectively.^35^ The fine structures written here show higher spatial resolution in all spatial dimensions. The observation of even smaller structures can be explained by the nonlinearity of multiphoton absorption in connection with the fact that broadening of the volume in which polymerization takes place is less than pronounced because diffusion is retarded by the gel network. In contrast, even some sharpening might be expected because of the in‐gel precipitation of the photopolymerized resin.

Figure 1e shows a comparison of the fragility and durability of in‐gel written helices that are supported by the gel structure and helices that were written into the liquid monomer. In the second case, the helix had to be fixed at a glass body and a self‐supported helix was only formed within a rather narrow window of size and crosslinking density of the resin, which is determined by the laser power employed per volume element. When the helix was mechanically weak, it collapsed upon drying. When it was too long, it collapsed under its own weight. In contrast, very long helices as well as mechanically weak helices could be inscribed into the gel. As a side effect, we observed bubble formation in the plain resin at laser powers exceeding 30 mW, while bubbles were not formed in the gels even at laser power as high as 50 mW. This can be partly explained by the fact that the initiator concentration within the gel is smaller than in the pure resin because the gel network occupies about 75% of the volume.

For the examples in Figure 1, the DLW conditions were chosen to generate a helix inside the gel with relatively high stiffness, that is, approaching the maximum curing of the IP‐L 780 resin. The in‐gel DLW, however, generates an interpenetrating network consisting of the hydrogel and the resin whereby the segregation of the cured IP‐L 780 resin is restricted to the sub‐micrometer scale or even smaller domains by the fact that the hydrogel network strands cannot move out due to their covalent network connectivity.^36^ Properties like the elasticity of such interpenetrating networks (IPNs) depend on the crosslinking of both constituent components and the extend of microsegregation. The crosslinking density in the interpenetrating network structure can be altered by the extent of photocuring of the IP‐L 780 resin. **Figure** **2** addresses this by summarizing results on differently photocured gel objects. The extent of curing depends on the intensity of the laser, the scanning speed, and the overlap between scan lines (Figure 2a). Very narrow distances between scan lines result in multiple exposure of the same volume and increased curing. The example in Figure 2b,c shows six differently irradiated rectangular, IP‐L 780 swollen gel patches (width:length: height = 10 µm × 30 µm × 5 µm) that are interconnected by nonirradiated fillets. A gradient in the degree of curing of the IP‐L 780 was generated by line scans from left to right that were advanced in lateral and vertical steps of 200 nm. The scan speed was kept constant at 5000 µm s^−1^ but the intensity was raised in 5 mW steps from 15 to 40 mW from left to right. Swelling in water demonstrates how the modulus of the IPN can be controlled by different slicing/hatching distance and laser power.^37^ Figure 2d demonstrates how writing conditions change the swelling property of gels (see also Figure S3, Supporting Information). The swelling of the samples was measured relative to the original rectangular photoresist swelled structures (5 µm × 50 µm × 5 µm) by the linear strain ε (%) = −(*h*_0_ − *h*)/*h*_0_ × 100, where *h*_0_ is the initial length and *h* is the length in water. It is important to notice that the samples in Figure 2d swelled homogenously but to a different extent on the scale of the image resolution although the weakly irradiated sample in Figure 2d, (ii) appeared pixeled. PNIPAm gels in which no photoresist was inscribed, swelled in water to a linear strain ε of 70% strain and collapsed by heating to a strain ε of 30% (Figure 2e). The structures into which the resin was inscribed with low power and thus at low density (300 nm, 20 mW) displayed nearly the same swelling. When the conversion of the resin was increased by more narrow slicing/hatching distance and higher laser power, swelling in water and consequently the collapse at elevated temperature was decreased. The formation of a second IPN did, however, not affect the transition temperature of the PNIPAm hydrogel component (LCST ≅ 32 °C)^4^ but only the extent of volume change. The observation that the expansion was reduced but the volume phase transition still occurred at the same temperature indicates phase segregation of PNIPAm and the polyacrylic resin, so that the swelling is constrained but the intrinsic PNIPAm properties remain unaffected.

**Figure 2:**
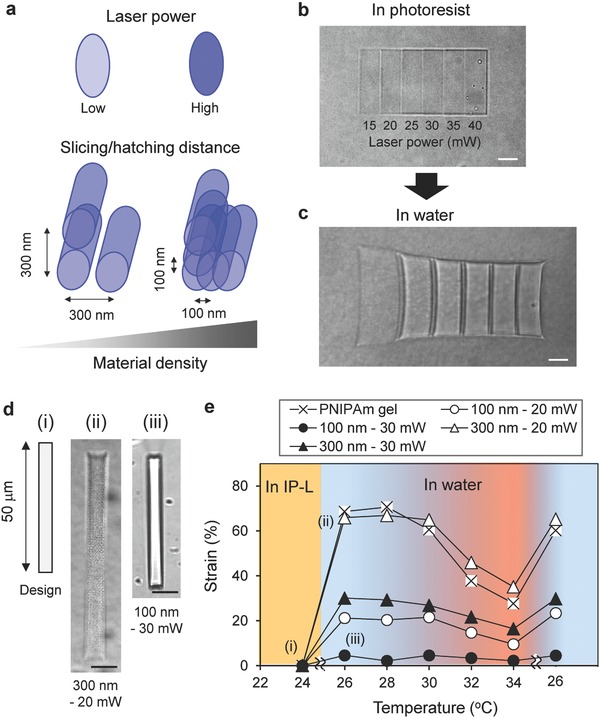
a) Laser power in focal spot and slicing/hatching distance determine the material density of the structure of IP‐L 780. b,c) Phase contrast images of a stiffness‐gradient structure swelled in photoresist (10 µm × 30 µm × 5 µm) and in water, respectively. The structure was composed of the compartments prepared by different laser power from 15 to 40 mW. d) Swelling of a rectangular stripe (5 µm × 50 µm × 5 µm) in water in which the resin was inscribed with varied laser power (20 and 30 mW) and slicing/hatching distance (100 and 300 nm, phase contrast images). e) Temperature and solvent dependence of the degree of swelling in water for different curing of the resin. The linear strain is given relative to the dimensions of the resin swollen PNIPAM before extraction and swelling with water (ε (%) = −(*h*_0_ − *h*)/*h*_0_ × 100, where *h*_0_ is initial length). Each strain value denotes an average of three different sample. Scale bars: 20 µm for (b)–(d).

The purpose of writing a skeleton structure into a hydrogel object is to direct the volume change upon uptake and release of water to controlled bending and distortion modes. This is demonstrated in **Figure** **3** by two examples. In the first case, parallel slats were inscribed into a hydrogel disc with a diameter as prepared diameter of 100 µm and a height of 5 µm. To explore the scope of anisotropic self‐shaping, three different slat patterns were inscribed into the disc shaped gel: (i) fully cured lamellae with a lateral thickness of 5 µm, a lateral interspace of 5 µm, and a height *h* = 5 µm, spanning the thickness of the disc from bottom to top (Figure 3a); (ii) a gradient pattern of the same geometry but with slats whose degree of curing and rigidity was gradually changed from one pole to the other pole of the disc (Figure 3b); and (iii) a similar gradient pattern with slats that did not pierce the disc from top to bottom and were thus positioned to the top side only (Figure 3c). Because the slats were inscribed into the resin swelled disc with a degree of swelling of 30%, they remained flat discs when they were brought into water at a temperature above the phase volume transition where the swelling by water is similarly small. At lower temperatures, however, when the PNIPAm swelled strongly all three objects demonstrated distinct buckling. In the first case an ellipsoid dome was formed elongated in the direction perpendicular to the lamellar and with gradual bending perpendicular to both main axis. When the same pattern was inscribed with a stiffness gradient a conical dome resulted with gradients in bending and stretching of the gel that were controlled by the stiffness of the slats. For the third case, we observed the most complex deformation when the disc rolled up to form a well‐defined cone. As seen in pine cone, anisotropic, different swelling patterns in bilayer structure result in curving of the structure with different curvature. Figure 3d depicts the designed shape variation by plotting the projection of the 2D perimeter. These data support that each patterning induced programmed 3D patterns including anisotropic swelling, asymmetric shape, and rolling up, while as‐prepared microgels show isotropic swelling behavior (Figure S4b, Supporting Information).

**Figure 3:**
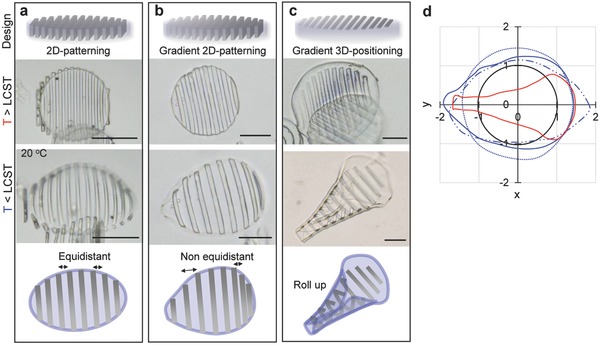
a–c) Schematic illustration of design of patterning in PNIPAm microgels and phase contrast images of each microgel in water at 20 °C. The 2D‐patterning denotes thick line pattern (*h* = 5 µm) cross‐linked from top to bottom of microdisc by 20 mW of laser power like conventional 2D lithography (2D patterning). The gradient 2D patterning has stiffness gradient by changing laser power 20 to 40 mW. The 3D positioning denotes thin line pattern (*h* = 500 nm) cross‐linked only the top part with stiffness gradient. d) Comparison of the area of microgels from top of view when the radius of each microgel at 32 °C was set to 1. Pure PNIPAm microgels (dot), 2D patterning (dot and line), gradient 2D patterning (blue line), gradient 3D patterning (red line), and microgel at 34 °C (black line). Scale bars, 50 µm.

In order to demonstrate even more complex shape variations, a conical spiral structure was inscribed into a gel disc as shown in **Figure** **4**a (radius of the spiral at the top and bottom was φ = 1 and 50 µm, respectively, and the pitch was 500 nm). Upon expansion of the gel, the helix generated a 3D nonuniform stress and the disc folded up to a flower‐like structure (Figure 4b). Figure 4c shows a confocal microscopy image with the fluorescence generated by the IP‐L‐780 resin. A cone‐like 3D shape was formed as the swelling gel expanded the spiral. The height of the spiral increased from 3 to 40 µm. When the temperature was raised, the disc relaxed first to the flat state and finally rolled up in a similar way like the object in Figure 4d, as the hydrogel contracted further. The data in Figure 4 are first and crude examples of how the origami‐like buckling and folding can be controlled by the in‐gel DLW techniques. Further examples are given in the Supporting Information including unfurling by favoring bending around one axis only, more complex wrinkling by crossed ribbons at one surface only, and a combination of wrinkling and bending (Figure S5 and Video S1, Supporting Information).

**Figure 4:**
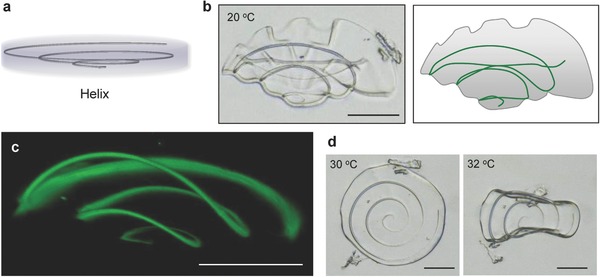
a) Design of a helix inscribed in microgel. b) Phase contrast image and schematic illustration of flower‐like microgel where helical structure (φ = 1 and 50 µm, pitch = 500 nm) was written when swelled in water at 20 °C. c) 3D‐reconstructed CLSM image of helix written in microgels swelled in water. d) Thermal actuation of microgel when temperature was increased over LCST. The microgel transformed the shape to flat at 30 °C and flapped at 32 °C. Scale bars, 50 µm.

In conclusion, the in‐gel DLW procedure we presented here offers some not directly foreseen advantages. This process shows the high resolution by which the structures can be inscribed and the interpenetrating way by which the two materials, resin and hydrogel, can be combined, so that microphase segregation occurs only at small distances, that is, below the length scale of the wavelength of light, while at the same time, the constituent materials retain their properties. The 3D position and mechanical properties of the inscribed structures could be tailored with a resolution better than 100 nm and a wide range of density. Here DLW offers nearly unlimited freedom in the design of the inscribed structures. These points enabled us to inscribe freely suspended microstructures of a second material like a skeleton into the hydrogel body and to direct volume changes of the hydrogel gel to controlled bending and distortion motions. In the combination with a hydrogel that undergoes a volume phase transition rather small temperature variations could actuate large amplitude motions. This allowed us to generate complex modes of motion through the rational engineering of the stresses that are generated in the multicomponent material. We consider the examples shown here as a beginning regarding the design of the structures, but also the variation of chemical compositions. In principle, the gel bodies can be swelled and inscribed even by different materials in a multistep procedure. More sophisticated designs can realize multiple, programmable actuations toward biomimetic soft robots. Furthermore, the modification of hydrogel by the micro/nanofabrication offers the possibility to develop adaptive, reversible photonic materials. Encoding of periodic nanostructure to gels provides photonic materials that can tune their reflectance and color in response to external stimuli, which leads to data encryption, sensor, and display device.^35^

## Supporting information

Supplementary

Supplementary
