# Supplementary material for: In‐Gel Direct Laser Writing for 3D‐Designed Hydrogel Composites That Undergo Complex Self‐Shaping
Source: Adv Sci (Weinh). 2017 Jul 25;5(1):1700038. doi: 10.1002/advs.201700038 (PMC5770688; doi:10.1002/advs.201700038)
Supplement: Supplementary file 1 — Supplementary [file ADVS-5-na-s001.pdf]

Copyright WILEY-VCH Verlag GmbH & Co. KGaA, 69469 Weinheim, Germany, 2017.

ADVANCED SCIENCE
Open Access

Supporting Information

for *Adv. Sci.,* DOI: 10.1002/advs.201700038

In-Gel Direct Laser Writing for 3D-Designed Hydrogel Composites That Undergo Complex Self-Shaping

*Akihiro Nishiguchi, Ahmed Mourran, Hang Zhang, and Martin Möller**

Supporting Information should be included here (for submission only; for publication, please provide Supporting Information as a separate PDF file).

Supporting Information

In-Gel Direct Laser Writing for 3D-Designed Hydrogel-Composites that Undergo Complex Self-Shaping

By *Akihiro Nishiguchi, Ahmed Mourran, Hang Zhang, and Martin Möller**
Dr. Akihiro Nishiguchi, Dr. Ahmed Mourran, M.Sc. Hang Zhang, [*] Prof. Dr. Martin Möller
DWI Leibniz-Institute for Interactive Materials, and Institute of Technical and Macromolecular Chemistry, RWTH Aachen University
Forckenbeck str. 50, D-52056, Aachen (Germany)
E-mail: moeller@dwi.rwth-aachen.de,

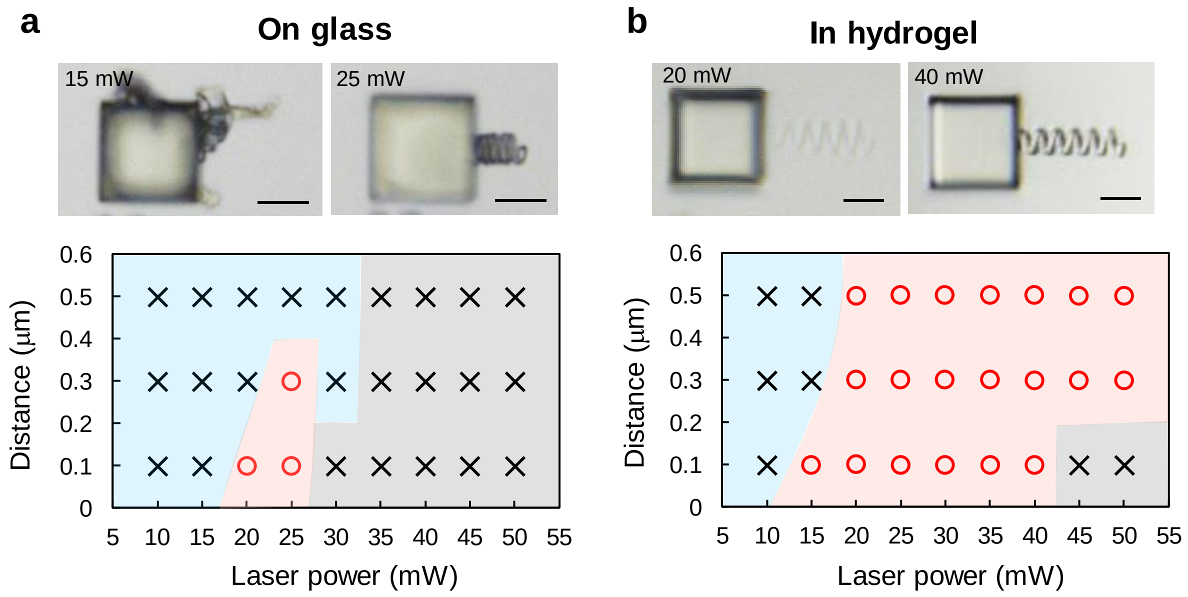

Figure S1. Phase contrast images and shape diagram of 20 μm length of helices written (a) on a glass and (b) in hydrogel. The laser power and slicing/hatching distance were varied from 10 to 50 mW and from 100 to 500 nm, respectively. Scan speed was fixed at 5000 μm s^{-1}. Circle, cross, and triangle marks denote structure formation, no structure, and bubble formation, respectively. Scale bars, 10 μm.

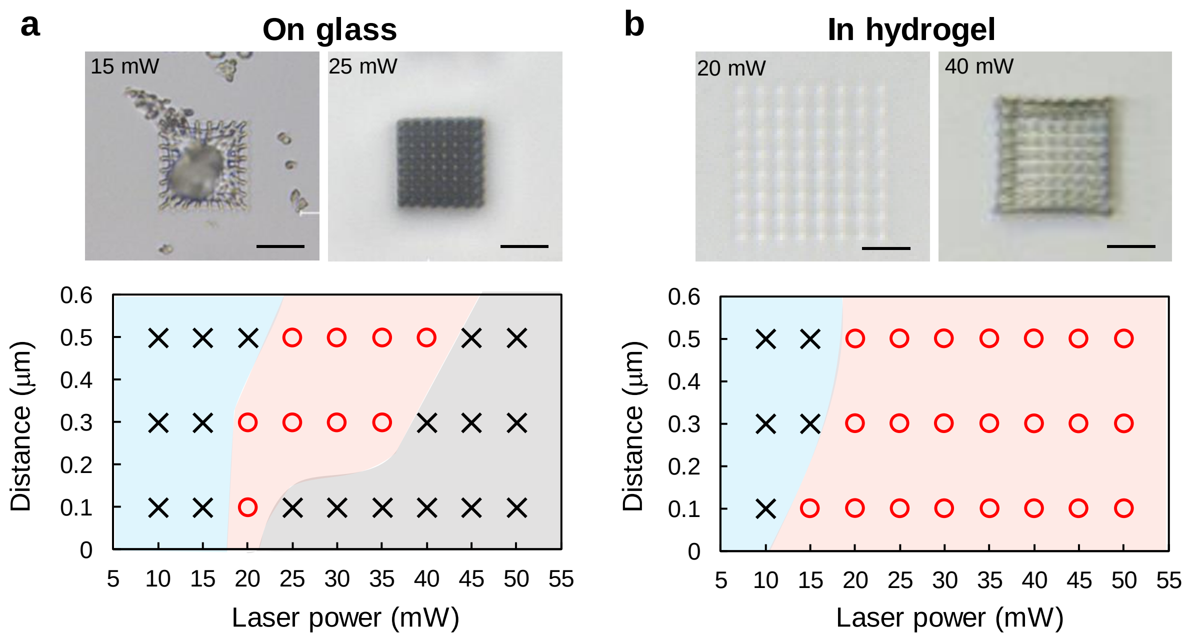

Figure S2. Phase contrast images and shape diagram of 20 μm x 20 μm x 20 μm of grid structure written (a) on a glass and (b) in hydrogel. The laser power and slicing/hatching distance were varied from 10 to 50 mW and from 100 to 500 nm, respectively. Scan speed was fixed at 5000 μm s^{-1}. Circle, cross, and triangle marks denote structure formation, no structure, and bubble formation, respectively. Scale bars, 10 μm.

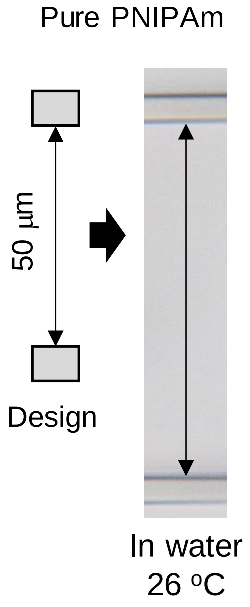

Figure S3. Schematic illustration and phase contrast images of as-prepared PNIPAm gel without structure in between (pure PNIPAm).

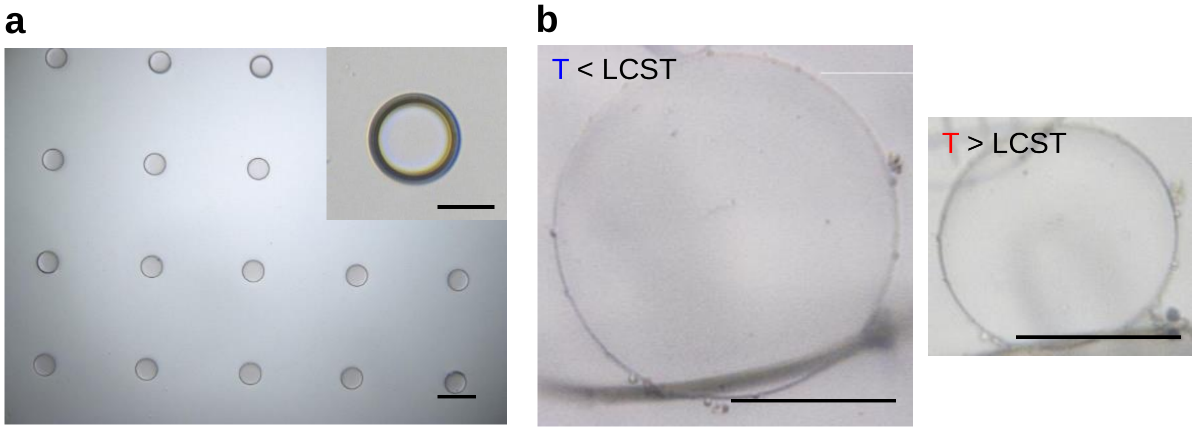

Figure S4. (a) Phase contrast images of array of microdisc of PNIPAm gel on a PFPE mold. (b) Phase contrast images of microgels after collecting from a substrate and swelling in water below or above LCST, respectively. Scale bars, 50 μm (a, b), 20 μm (inset in a).

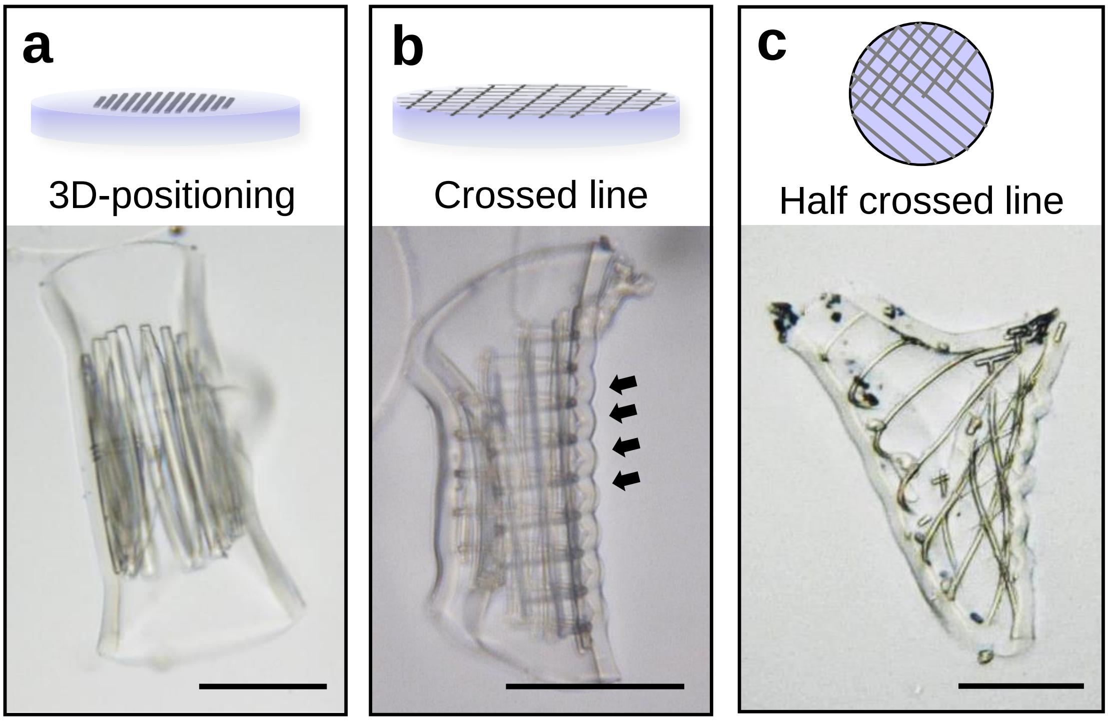

Figure S5. Schematic illustration of design and phase contrast images of each microgel in water at 20 ^{o}C. a) Thin line patterns (h=500 nm) written in the middle of microgels. b) Crossed lines prepared on microgels and attached with one part of crossed lines. c) Half crossed lines prepared on microgels. Scale bars, 50 μm (a, b) and 20 μm (c).

Movie S1. Flower-like microgel actuation in response to temperature (20 to 32 ^{o}C and then 32 to 20 ^{o}C, x16 speed).
